# Establishing reference values for macro- and microvascular measurements in 4-to-5 year-old children of the ENVIR*ON*AGE prospective birth cohort

**DOI:** 10.1038/s41598-020-61987-z

**Published:** 2020-03-20

**Authors:** Narjes Madhloum, Leen J. Luyten, Eline B. Provost, Patrick De Boever, Yinthe Dockx, Hanne Sleurs, Michelle Plusquin, Jos op’t Roodt, Karen Vrijens, Tim S. Nawrot

**Affiliations:** 1 Centre for Environmental Sciences, Hasselt University Hasselt, Belgium; 2 Unité de Recherche en Biologie Cellulaire (URBC) - Namur Research Institute for Life Sciences (Narilis), Namur University, Namur, Belgium; 3 Health Unit, Flemish Institute for Technological Research (VITO), Mol, Belgium; 4 Department of Internal Medicine, Maastricht University Medical Centre (MUMC+), Maastricht, The Netherlands; 5 Department of Public Health & Primary Care, Occupational and Environmental Medicine, Leuven University, Leuven, Belgium

**Keywords:** Environmental sciences, Biomarkers

## Abstract

Cardiovascular risk factors are usually better tolerated, and can therefore be perceived as less harmful, at a young age. However, over time the effects of these adverse factors may persist or accumulate and lead to excess morbidity and mortality from cardiovascular diseases later in life. Until now, reference values for the basic cardiovascular health characteristics of 4-to-6 year-old children are lacking. Within a follow-up study of the ENVIR*ON*AGE (ENVIRonmental influence *ON* early AGE) birth cohort we assessed various cardiovascular measurements in 288 children aged 4–5 years. For the macrovasculature, we measured their blood pressure and examined the intima-media thickness of the carotid artery (CIMT), the arterial elasticity (including the pulse-wave velocity (PWV), carotid distensibility (DC) and compliance (CC) coefficients), the carotid β stiffness index (SIβ) and Young’s Elastic Modulus (YEM). Retinal microvascular traits included the Central Retinal Arteriolar Equivalent (CRAE) and Central Retinal Venular Equivalent (CRVE). Age of the study population averaged (±SD) 4.2 (±0.4 years. Mean systolic and diastolic blood pressure were 97.9 (±8.1) mmHg and 54.7(±7.6) mmHg, respectively. CIMT for the total population averaged 487.1 (±68.1) µm. The average stiffness values for DC, CC, SIβ, and PWV were 78.7 (±34.2) 10^−^³/kPa, 1.61 (±0.59) mm^2^/kPa and 4.4 (±2.4), and 3.7 m/s (±0.9) respectively. The mean determined for YEM was 163.2 kPa (±79.9). Concerning the microvasculature, the average CRAE was 180.9 (±14.2) µm and the corresponding value for CRVE was 251.0 (±19.7) µm. In contrast to the macrovasculature, a significant gender-related difference existed for the microvasculature: in boys, both the CRAE (178.8 µm vs 182.6 µm; p = 0.03) and CRVE (247.9 µm vs 254.0 µm; p = 0.01) were narrower than in girls. We have provided reference values for young children to understand changes in the early cardiovascular health trajectory. Establishing these reference values of cardiovascular phenotypes at this young age is necessary to develop targeted health promotion strategies as well as for better understanding of the life course changes of both small and large blood vessels.

## Introduction

Risk factors for the development of cardiovascular diseases, such as smoking, an inactive lifestyle, and high blood pressure are usually better tolerated at a younger age and are therefore often perceived as less harmful within this phase in life. However, if these physical challenges persist over time, they may lead to an increase in morbidity and mortality from cardiovascular causes in midlife. In adults, several clinical parameters have been linked to cardiovascular disease trajectories. For example, the distance between the intima and media layer of the carotid vessel wall is commonly used as an indicator of the extent of atherosclerosis and the chance of developing cerebral and cardiovascular incidences^1^. For adults at risk of diseases of the heart and the circulatory system, guidelines for the primary prevention of these conditions have been well documented^2^. Cardiovascular diseases often find their origin during childhood. Atherosclerotic lesions, which are well-known as a subclinical biomarker for endothelial dysfunction, begin to form in early in life with the accumulation of fatty streaks^3^. An autopsy study in children demonstrated that nearly all children have at least some degree of aortic fatty streaks by the age of three, with atherosclerotic lesions present in the coronary arteries during adolescence^4^. Establishing reference values of cardiovascular phenotypes early in life before the first signs of atherosclerosis appear is equally important and necessary to develop targeted health promotion strategies as well as to get a better understanding of the life course changes of both small and large blood vessels and early cardiovascular health trajectories within the Developmental Origins of Health and Disease (DOHaD). Untill now, very few population-based studies have been conducted that evaluate overall cardiovascular health and macrovascular stiffness markers in young children under the age of eight^5–7^. Long-term studies on the microvasculature are needed as well, to assess the prediction of its reactivity^7^ and function throughout childhood into adolescence.

In this study, we provide reference values for both macrovascular and microvascular characteristics in 4-to-5-year-old children. For the macrovasculature, references have been determined for the carotid artery intima-media thickness (CIMT) and the arterial stiffness parameters, including the pulse-wave velocity (PWV), carotid distensibility (DC) and compliance coefficients(CC), the carotid stiffness β index (SIβ) and Young’s Elastic Modulus (YEM) and blood pressure (BP). Microvascular measurements are based on retinal vessel analysis using fundus photography, different than other studies where microvascular measurements were performed by the assessment of skin microcirculation^6^, nail fold videocapillaroscopy^7^ or by recording the pulse wave amplitudes of fingertips^5^. Diameters of the central retinal vessels, summarized as the central retinal arteriole equivalent (CRAE) and central retinal venular equivalent (CRVE), were used as proxies.

## Methods

### Study cohort

The data were obtained from the follow-up study of the ENVIR*ON*AGE (ENVIRonmental influence *ON* early AGEing) birth cohort. This longitudinal cohort has been described in more detail previously^8^. In brief, ENVIR*ON*AGE is a population-based prospective birth cohort study located in Limburg, Belgium. Mothers were originally recruited at the time of birth of their child at the Hospital East Limburg in Genk, Belgium. At this moment the participants signed a first informed consent form and provided their contact details, for the purpose of contacting them to participate in later stages of the study. At 4-to-5 years of age, the children and their mothers were invited for cardiovascular phenotyping. The macrovascular measurements were performed in a quiet and comfortable environment in supine position after 5 minutes rest. The protocol of the ENVIR*ON*AGE birth cohort follow-up study was approved by the Ethics Committee of Hasselt University and was in compliance with the Declaration of Helsinki. This follow-up study was performed in accordance with relevant regulations and all clinical measurements were carried out following standardized guidelines. Participating mothers provided written informed consent and children gave their assent. Besides information on the general health and nutrition of the children, the medical history and smoking habits of the parents and grandparents were inquired with a questionnaire filled in by the mother. The section on smoking behavior questioned if one or both parents were smoking at the time of birth, in the first six months of life, the first year of life or until the moment of the follow-up visit and whether they smoke(d) in the presence of their child(ren). Before the measurements were initiated, it was always indicated by the examiners that, at any point during the clinical examinations, these could be stopped if the child was not or no longer willing to perform the tests.

**Figure 1:**
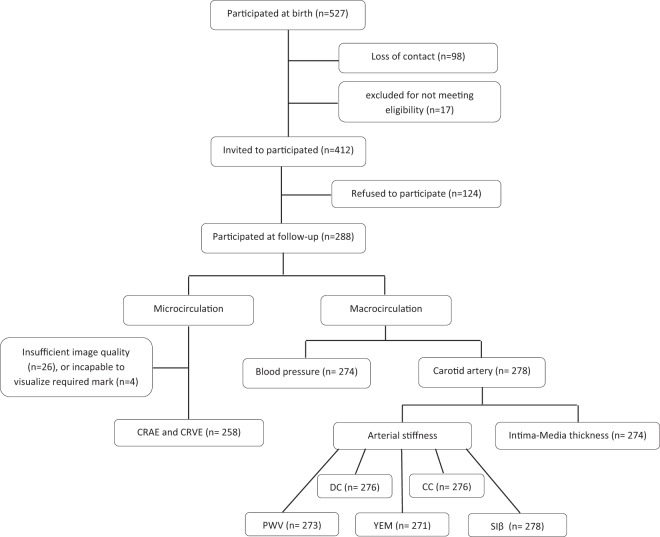
Flow chart showing the total number of measurements taken for macro- and micro-vascular values. CIMT = carotid artery intima-media thickness, PWV = the pulse-wave velocity, DC = carotid distensibility, CC = compliance coefficients, Siβ = the stiffness β index, YEM = Young’s Elastic Modulus, BP = blood pressure, CRAE = central retinal arteriole equivalent, CRVE = central retinal venular equivalent.

A total of 527 mother-child pairs, of which the children were born between February 2^nd^ 2010 and November 3^rd^ 2013, were contacted for the follow-up study. Participants were excluded if the mother was not able to fill in a questionnaire in Dutch (n = 3), if the children had medical conditions compromising their participation (n = 5), or passed away (n = 1) or moved out of the study area (n = 8). Of the remaining 510 participants, we were not able to reach 98 mothers due to loss of contact. One hundred and twenty-four women refused to participate in the study, the main reasons for refusal were: lack of time or no interest in contributing to this part of the study (n = 109), or the child having difficult behavior (n = 6). Of the 412 invited mother-child pairs, 288 agreed to participate in the study (Fig. 1). Thus the overall participation rate was 57%.

Background characteristics of the 222 non-participants were similar to participants with respect to: mean age of the mother (28.1 *vs* 30.0 years, respectively), sex distribution (110 [49.6%] *vs* 143 [49.5%] girls, respectively), and maternal education (low, medium, and high: 14 [6.3%], 61 [27.5%], and 147 [66.2%] *vs* 18 [6.2%], 79 [27.4%], and 191 [66.3%], respectively).

Clinical characteristics could be obtained from almost all participating children. Of the total study population, fourteen children refused to have their BP taken and ten children would not undergo the ultrasound CIMT measurement. In 26 children (9.4%) the CIMT could only be obtained by two measurements taken at two different angles and in two children (0.7%) only one measurement could be performed at one angle and one measurement could not be used due to bad quality caused by continuous movement of the child (n = 1). The stiffness parameters could not be calculated for all children due to refusal of the ultrasound measurement (n = 11) or due to poor quality of the final images (n = 5). For the microvasculature, the retinal blood vessel images of 30 participants could not be analyzed. Four children were incapable to visualize the required mark due to visual or mental restrictions. Of the remaining excluded images (n = 26), the quality was insufficient to perform the analyses, due to movement of the child’s eyes if they would not keep their head in a steady position or would not keep looking at the required mark to center the optic disk.

### Anthropometric characteristics

The height of the children was measured using a fixed stadiometer with an accuracy of 0.5 cm. Waist circumference was determined at the level of the umbilicus to the nearest 0.1 cm. The children’s body weight was measured to the nearest 0.1 kilogram with a digital scale and the body mass index (BMI) was calculated. The anthropometric characteristics were obtained as described by the WHO guidelines^9^.

### Cardiovascular phenotyping

#### Blood pressure

Children’s BP was measured with an automated oscillometric device (Omron 705IT, Omron Corporation, Japan). To ensure an accurate measurement, appropriate cuffs adapted for children, were used depending on the child’s right upper arm circumference, according to the recommendations of the American Heart Association^10^. The sizes were used small (15–22 cm) or xsmall (9–14 cm). Five consecutive measurements were taken with one-minute intervals according to a standardized protocol^10^. For our analyses, we used the mean systolic blood pressure (SBP) and mean diastolic blood pressure (DBP) averaged for the last three measurements. If there was a greater than 5 mmHg difference between the last three readings, one additional measurement was performed.

#### Ultrasound imaging/arterial wall parameters

##### Carotid intima-media thickness and distention measurements

Imaging of the right carotid artery was performed by a single trained investigator (NM). The ultrasound technique was equipped with automated boundary detection software in highly accurate radio frequency (RF)-data acquisition and a vascular probe at 13 MHz (MyLabOne, Esaote Benelux, Maastricht, The Netherlands)^11^. Longitudinal scanning of a ≥ 1 cm segment of the common carotid artery measured the distance between the luminal border of the intima and the outer border of the media of the carotid artery far wall. This distance is represented as a double-line pattern on a B-mode ultrasound image, at 1–2 cm proximal to the dilatation of the carotid bulb. CIMT was determined under three different angles; i.e. 90°, 130° and 180° relative to the direction of the carotid artery, using Meijer’s Arc. Prior to the ultrasound measurements, the participants rested for 10 min in a supine position, with the head lightly hyperextended and rotated to the left in the direction opposite the probe. The CIMT measurements were obtained and evaluated online. However, in a later phase (off-line), the ultrasound images were double-checked for quality by a highly experienced vascular expert (JoR) blinded for the study and certified from the division of the vascular ultrasound research.

##### Carotid stiffness

The arterial stiffness parameters were obtained with the same method and device as used for the CIMT measurements. Carotid stiffness describes the rigidity of the arterial wall and can be assessed in different ways. The stiffness parameters were described as the carotid distensibility coefficient (DC) and compliance coefficient (CC), pulse wave velocity (PWV) and the stiffness index β (SIβ). These values were derived from the ultrasound measurements averaged over eight cardiac cycles with a duration of 6 seconds, which measures the lumen diameters during peak systole and end diastole. The brachial blood pressure was measured before the ultrasound examination and was entered to be taken into account during the ultrasound.

The DC, measured as changes in arterial diameter or circumferential area in systole and diastole, is a reflection of the mechanical stress affecting the arterial wall during the cardiac cycle and is formulated as DC = (D_s_ − D_d_/D_d_)/(P_s_ − P_d_). The CC, the change in diameter of the artery in response to a given change in expanding pressure, was determined with the formula: CC = (D_s_ − D_d_)/(P_s_ − P_d_). The local PWV can be defined as the speed of travel of the pressure pulse wave along a specified distance along the vascular bed, according to PWV = 1/√ρ×DC, with ρ as blood density. The DC and CC are inversely related to arterial stiffness while PWV is a direct measure of this vessel characteristic. The SIβ, another direct marker of arterial stiffness, reflects functional damage to the vasculature and is formulated as SIβ = ln(P_s_/P_d_)/[(D_s_ − D_d_)/D_d_]. In addition, we computed the Young’s Elastic Modulus (YEM), a parameter that combines measures of arterial wall elasticity with wall thickness and is calculated as YEM = (SBP-DBP) * D_d_/(D_s_ − D_d_) * IMT with SBP = systolic blood pressure, DBP = diastolic blood pressure, Ds = end-systolic diameter of the artery and Dd = end-diastolic diameter of the artery, P_s_ = is systolic blood pressure, P_d_ = diastolic blood pressure. An increase in YEM represents an increase in arterial stiffness.

#### Retinal vessel diameters

Images of the fundus of both the left and right eye were taken with a Canon CR-2 plus 45° 6.3 megapixel digital nonmydriatic retinal camera (Hospithera, Brussels, Belgium). Retinal vessel diameters were determined with the retinal vessel analysis MONA software (version 2.1.1) developed at VITO (Mol, Belgium). Central retinal arteriolar equivalent (CRAE) and central retinal venular equivalent (CRVE) were calculated between a distance of 0.5 and 1 times the optic disc diameter^12^. For this purpose, the average width of respectively the six largest arterioles and six largest venules was calculated using the revised Parr-Hubbard formulas^13^. The average CRAE and CRVE could be calculated from the values of both eyes for 205 out of the 258 children for which pictures of sufficient quality were available. For 53 children, only the measurements of one eye could be used (for 31 participants only the left eye, for 22 children only the right eye). Subsequently, these outcomes were used for the statistical analyses within this study population. One grader (LL) scored all images.

### Statistical analysis

We used SAS software (version 9.4; SAS Institute Inc., Cary, NC, USA) for database management and statistical analyses. Data are presented as averages with their respective standard deviation (SD), and upper (75^th^ percentile) and lower (25^th^ percentile) limits. Categorical data are presented as frequencies (%) and associated number of participants. Normality of the data was tested with the Shapiro-Wilk test. Correlation of variables was determined with Pearson correlation coefficients. An independent t*-*test was performed to compare averages between the boys and girls within our study population.

## Results

### Study cohort

The anthropometric measurements of this follow-up reference cohort are characterized in Table 1. A total of 288 children (49.7% girls) were enrolled in the study. Eighty-seven percent of the children were at age 4 at the moment of the follow-up study visit, 11.8% were at age 5 and one child was 6 years old. The children had an average height of 107.9 (±4.9) cm and an average weight of 18.8 (±2.8) kg, the gender-specific anthropometric variables are given in Table 1. The average BMI was equal for the total population as well as for boys and girls separately, namely 16.1 (±1.5) kg/m². Most of the children (96.5%) had no medical condition and were in good health. Of the remaining children three had asthma, two had a fruit allergy, one child had an immune disorder, two had epilepsy and two children had a congenital bone developmental disorder. Eighty-two parents (28.4%), stated to smoke regularly, of which 43 parents (14.9%) smoked in the presence of their child. The SIβ (r = 0.29; p = 0.035) was positively correlated among children whose parents smoked in their direct vicinity. A history of cardiovascular disease was present in 38% of the grandparents and 4.9% of the parents. Thirteen percent of the fathers and 6.6% of the mothers had a BMI above 30 kg/m³. We did not observe any correlations between family history (high blood pressure or other cardiovascular diseases) and the macrovascular measurements of the children. There were no significant differences in personal and general health characteristics between boys and girls. Percentiles (5^th^, 10^th^, 50^th^, 90^th^ and 95^th^) tables for each population characteristics are presented in Table S1 for the total study population and Table S2 for boys and girls separately.”

**Table 1:** Characteristics of the follow-up study population showing the number of participants for each parameter, the average ± standard deviation (SD) and the 25th (P25) and 75th (P75) percentile.

	Total				Boys				Girls			
	N	Mean (SD)	P25	P75	N	Mean (SD)	P25	P75	N	Mean (SD)	P25	P75
**Personal characteristics**												
Age, years	288	4.2 (0.4)	4.3	4.8	145	4.4 (0.4)	4.3	4.8	143	4.3 (0.4)	4.3	4.7
Height, cm	288	107.9 (4.9)	104.3	111.0	145	109.3 (4.9)	104.4	111.0	143	105.5 (5.1)	104.3	110.5
Weight, kg	288	18.8 (2.8)	17.0	20.3	145	19.7 (2.9)	17.2	20.3	143	19.6 (2.8)	17.0	20.3
BMI, kg/m²	288	16.1 (1.5)	15.1	16.9	145	16.5 (1.5)	15.2	16.9	143	17.2 (1.5)	15.0	16.9
**Macrovascular characteristics**												
Systolic blood pressure, mmHg	274	97.9 (8.1)	93.4	102.3	142	103.9(8.3)	93.3	102.3	132	97.0 (7.8)	93.9	101.7
Diastolic blood pressure, mmHg	274	54.7 (7.6)	50.3	58.3	142	71.0 (8.3)	49.1	57.3	132	63.0 (6.7)	51.0	59.3
Heart rate, beats per minute	274	89.8 (11.1)	83.7	96.7	142	52.2 (12.1)	84.7	96.3	132	98.3 (9.8)	82.9	96.8
Carotid intima-media thickness, µm	274	487.1 (68.1)	433.9	525.8	138	479.0 (69.8)	441.0	543.3	136	483.1 (67.0)	432.6	521.6
Pulse wave velocity, m/s	273	3.7 (0.9)	3.19	4.08	135	3.2 (0.9)	3.1	4.1	138	3.2 (0.8)	3.2	4.1
Distensibility coefficient,10^−23^/kPa	276	78.7 (34.2)	58.8	94.4	137	105.6 (38.4)	54.1	96.3	139	83.3 (29.5)	61.8	91.5
Young’s elastic modulus, kPa	271	163.2 (79.9)	111.2	186.7	134	122.1 (86.6)	108.0	196.2	137	99.0 (72.9)	112.3	183.6
Carotid distension, µm	278	766.6 (164.9)	661.0	881.0	138	753.0 (172.6)	662.5	889.3	135	828.8 (152.9)	645.0	862.0
Stiffness index β	278	4.4 (2.4)	3.2	4.8	139	2.9 (2.7)	3.16	4.8	139	2.5 (1.9)	3.3	4.9
Compliance coefficient, (mm^2^/kPa)	276	1.6 (0.60)	1.2	1.9	137	2.1 (0.6)	1.18	1.9	139	1.8 (0.5)	1.32	1.9
**Microvascular parameters**												
Central retinal arteriolar equivalent, µm	258	180.9 (14.2)	171.3	191.1	120	178.8 (15.2)	169.6	187.9	138	182.6 (13.5)	172.9	191.9
Central retinal venular equivalent, µm	258	251.0 (19.7)	238.5	264.3	120	247.9 (20.5)	235.0	259.4	138	253.9 (18.2)	240.8	268.0

### Blood pressure

Blood pressure measurements are presented in Table 1. Average SBP and DBP for the total study population were 97.9 (±8.1) mmHg and 54.7 (±7.6) mmHg, respectively. The SBP and DBP for boys averaged 103.9 (±8.3) mmHg and 71.0 (±8.3) mmHg and for girls 97.0 (±7.8) mmHg and 63.0 (±6.7) mmHg, respectively. No significant differences were noted between both sexes. DBP showed a positive correlation with CIMT (r = 0.19; p = 0.002) but was inversely associated with PWV (r = −0.14; p = 0.02). SBP correlated positively with YEM (r = 0.26; p = <0.0001) and PWV (r = 0.22; p = 0.0002).

### Macrovascular phenotypes

The average CIMT for the total study population was 487.1 (±68.1) µm. CIMT was measured in 138 boys and 136 girls and averaged 479.0 (±69.8) µm and 483.1 (±67.0) µm, respectively. The data showed a Gaussian distribution for both sexes. No significant differences were observed between boys and girls. CIMT measurements under the three different angles, i.e. 90, 150 and 180°, were moderately correlated (r = 0.56, p < 0.0001; 0.35, p < 0.0001 and 0.46, p < 0.0001 respectively). Stiffness parameter characteristics are described in Table 1. The average PWV, DC, CC, SIβ and YEM were 3.7 (±0.9) m/s, 78.7 (±34.2) 10^−^³/kPa, 1.6 (±0.59) mm^2^/kPa, 4.4 (2.4) and 163.2 (±79.9) kPa respectively. In boys the PWV, DC, CC, SIβ and the YEM averaged (SD) 3.2 (±0.9) m/s, 105.6 (±38.4) 10^−^³/kPa, 2.1 (±0.6) mm^2^/kPa, 2.9 (±2.7) and 122.1 (±86.6) kPa. In girls these values were 3.2 (±0.8) m/s, 83.3 (±29.5) 10^−^³/kPa, 1.8 (±0.5) mm^2^/kPa, 2.5 (±1.90) and 99.0 (±72.9) kPa for PWV, DC, CC, SIβ and YEM respectively. No significant differences were observed between boys and girls. All stiffness parameters were positively correlated with one another.

### Retinal vessel diameters

Table 1 summarizes the values of the retinal vessel diameters, as proxy for the microvasculature. Pictures of sufficient quality for analysis could be obtained for 258 children, which is 92.8% of the total follow-up study population. More specifically, these were derived from 120 boys (47.3%) and 138 girls (52.7%). For the entire study population, the average value of the CRAE was 180.9 (±14.2) µm and the mean CRVE was 251.0 (±19.7) µm. The CRAE was slightly higher in girls than in boys (182.6 *vs* 178.8 µm; p = 0.03), which was also the case for the CRVE (254.0 *vs* 247.9; p = 0.01). CRAE decreased with higher SBP (r = −0.12; p = 0.07) and DBP (r = −0.19; p = 0.0036). No association was present for both the SBP (p = 0.70) and the DBP (p = 0.44) in correlation with CRVE. Finally, no association was found between CRAE and CRVE with the IMT and the stiffness parameters.

## Discussion

A Lancet commission recently proposed “*to put pressure*” on hypertension and cardiovascular risks by tracking the life course of these silent killers affecting quality of life of populations worldwide^14^. In line with this call to action, ENVIR*ON*AGE enables a life course approach in identifying reference values for cardiovascular measurements from the early phases of life. We have established novel information on cardiovascular reference values, which could be used as preclinical biomarkers in children aged 4 living in an affluent Western society. The results could have important implications, as they could form a clinical base for the exploration of further vascular alterations over time from childhood into adulthood. Since much effort and a great budget are being invested in the primary prevention of adverse cardiovascular conditions later in life, monitoring of the condition of the heart and blood vessels in early childhood could potentially reduce these costs and the need for intervention later in life^15^.

The greatest attempt to establish cardiovascular reference values in children has been on blood pressure. As early as 1977, with a revision in 1987, the National Heart, Lung, and Blood Institute in Maryland (USA) set a list of reference BP values for a cohort of American children^16^. Furthermore, to study and prevent the development of hypertension, findings of six West-European cohorts, combining the results of over 28.000 participants, were used to summarize the SBP and DBP for children aged four to nineteen^17^. For CIMT, one study by Baroncini *et al*.^18^ has described these values for a smaller group of children (n = 93) aged one to five. References for CRAE and CRVE in very young children have been published for the Strabismus, Amblyopia and Refractive Error Study in Singaporean Chinese Preschoolers (STARS) cohort. In this study, both types of retinal vessel diameters were summarized for 469 children aged four to six^19^. However, a study that combines reference values for the macro- and microvascular parameters measured within a cohort on children at the age of four has never been performed before to the best of our knowledge.

Although cardiovascular diseases and events barely occur in young children, alterations in the circulatory system and an increment in the risk of developing these detrimental conditions can already occur early in life and can develop surreptitiously before clinical symptoms become apparent during adulthood. Apart from genetic predisposition, factors such as obesity and diabetes occurring in the earlier phases in life are known to augment the chances of atherosclerosis and detrimental cardiovascular events during adulthood^20^. Carotid intima-media thickness is a marker of preclinical atherosclerosis, which is the underlying subclinical biomarker for cardiovascular disease. Autopsy studies have shown the first evidence that the atherosclerotic process begins in childhood with the accumulation of lipids in the arterial wall to form fatty streaks, establishing a strong association between cardiovascular risk markers and preclinical atherosclerosis^4,21^. These early vascular changes can be assessed by ultrasound imaging, a noninvasive and reliable technique used to asses both vascular anatomy and function. In adults, a large number of studies exist that have associated an increased CIMT with coronary artery diseases and the prediction of cardiovascular events, including stroke and myocardial infarction^22^. Additionally, several cardiovascular risk factors have been associated with CIMT, including diabetes mellitus, total cholesterol levels and smoking^23,24^. Prospective longitudinal studies have demonstrated strong associations between cardiovascular risk factors and CIMT, measured from childhood to adulthood^25–27^. However, CIMT assessments have mostly been used in children with existing risk factors for cardiovascular disease. Both structural and functional vascular changes, including an increased CIMT and increased stiffness were demonstrated in pediatric patients with familial hypercholesterolemia, obesity, type 1 diabetes mellitus and hypertension^21,28^. In this context, high BP is known to be a major risk factor for cardiovascular disease and stroke^29^ and it is considered as an independent risk factor for an increased CIMT in young adulthood^26,30^. Consequently, high blood pressure in childhood could lead to hypertension in adulthood^31^. These findings have been confirmed by two longitudinal studies, resuming that BP trajectories in childhood may identify hypertension later in life, and this by enhancing two repeated observations of abnormal high blood pressure taken over several years^32,33^. Several studies reported that morphological and functional aspects of the common carotid artery are particularly influenced by age, body dimensions, and BP starting form young age^34–36^. In 1151 children aged 6–18 years old, investigating the association between CIMT and distensibility and have found an increased CIMT and decreased distensibility with age, height, body mass index, and BP^34^. Population-based studies in adults show that IMT and arterial stiffness increase over the life course^37,38^. Age- and sex specific differences in CIMT and distensibility were significant from the age of 15 years in a population of children aged 6–18 year old. In a 21-year follow-up of the Cardiovascular Risk in Young Finns Study^39^, elevated SBP in adolescence (at age 12 to 18 years) predicts endothelial dysfunction in adulthood. However, childhood (age 3 to 9 years old) SBP did not correlate with adult endothelial dysfunction^39^. Although, ageing is a main determinant in influencing the CIMT and endothelial wall^37^, we could not perform this association due to young age and the limited variability in age in our study population. Children (ages 11 to 14 years old) with high BP, showed a significant 11% higher PWV (4.91 ± 0.39 m/s) compared to children with normal BP (4.43 ± 0.32 m/s, p < 0.001)^40^. Furthermore, overweight and obesity are related to cardiovascular risk factors leading to early atherosclerosis in adults^41^ and young children^42,43^. In a group of 306 children with a similar age to our study population, an increased waist circumference was related to thicker and stiffer arteries^42,43^. In addition to body composition, second hand smoke is an important risk factor for arterial alterations from early life onwards as observed here and these effects might be long-lasting^44^.

Changes in the coronary macro- and microcirculation parallel those in the retinal microvasculature. Microcirculation comprises all blood vessels with a diameter equal to or smaller than 150 µm and makes up a large part of the total circulatory surface. A healthy and proper functioning microcirculation is crucial to supply tissues and organs with a sufficient amount of oxygen and substances to maintain the metabolism and for the removal of waist substances such as carbon dioxide^45^. Although these small vessels make up the bulk of the circulatory system, their role in the pathogenesis of age-related diseases is much less explored than that of the macrocirculation^46^. Microvascular alterations can be early markers of disease, secondary to the pathological process itself^47^. Although CRAE could be a potential clinical marker for cardiovascular disease, the exact relationship between blood pressure and changes in the retinal microvasculature can only be established in future follow-up research within this prospective cohort study.

### Gender-dependent differences in micro- but not macrovasculature

In this study, we did not observe any gender-dependent differences in blood pressure, IMT or stiffness parameters. This could suggest that differences in the regulation of blood pressure between men and women only become apparent at later stages in adult life under the influence of hormones^48^. A significant sex difference in these mascrovascular parameters was reported to become apparent from the age of 15 years^34^. In contrast to the macrovasculature, we did find a significant gender-dependent difference for both the arteriolar and venular diameter of the retina. The diameters of both retinal vessel types are smaller in boys than in girls in our follow-up cohort. In a study of Schlager *et al*.^49^, differences in the microcirculation of children, measured via Doppler on the skin of the hand, could only be observed between the age of 10 and 18 years old, but not at the age of 4.

### Strengths and limitations

One of our major strengths is that we were able obtain a wide panel of cardiovascular measurements in 4-to-5-year-olds using a standardized protocol and multiple measurements (five blood pressure assessments, CIMT determined at three different angels and retinal microvascular measures from both eyes). The ENVIR*ON*AGE study is a prospective birth cohort and is transferable to the general population as our study population is at baseline representative of the gestational segment of the population at large^8,50^ and at the age of 4 because of the high participation rate (57%) towards follow-up. As with all longitudinal studies we were not able to contact a part of the initial cohort (n = 98), however results are unlikely to be confounded by selection bias; participants and non-participants had similar sociodemographic characteristics such as sex, age, and maternal education.

## Conclusions and Future Directions

We have examined numerous cardiovascular characteristics of children at the age of four. With this, we have established potential reference values to map early changes of the circulatory system and to understand the trajectory of arterial stiffness and microvascular changes in an early phase of life. Establishing reference values of cardiovascular phenotypes at this early period in life is of great importance not only for better understanding of life course changes of small and large blood vessels and the subsequent consequences for the development of cardiovascular diseases such as atherosclerosis, but also for the realization of targeted health promotion strategies.

For the future of this follow-up cohort, we aim to continue our research on the more than 1000 remaining mother-child pairs of the ENVIR*ON*AGE birth cohort to confirm and further establish our results on the circulatory system.

## Supplementary information

Supplementary information.

## Data Availability

The datasets generated during and/or analysed during the current study are available from the corresponding author on reasonable request.
