# Supplementary material for: Establishing reference values for macro- and microvascular measurements in 4-to-5 year-old children of the ENVIRONAGE prospective birth cohort
Source: Sci Rep. 2020 Mar 20;10:5107. doi: 10.1038/s41598-020-61987-z (PMC7083909; doi:10.1038/s41598-020-61987-z)
Supplement: Supplementary file 1 — Supplementary information. [file 41598_2020_61987_MOESM1_ESM.pdf]

Supplemental material

Establishing reference values for macro- and microvascular measurements in 4-to-5 year-old children of the ENVIR*ON*AGE prospective birth cohort

Narjes Madhloum[1], Leen J. Luyten[1,2], Eline B. Provost[1,3], Patrick De Boever[1,3], Yinthe Dockx[1], Hanne Sleurs[1], Michelle Plusquin[1], Jos op't Roodt[1,4], Karen Vrijens[1], Tim S. Nawrot[1,5*]

[1]Centre for Environmental Sciences, Hasselt University, Hasselt, Belgium.
[2]Unité de Recherche en Biologie Cellulaire (URBC) - Namur Research Institute for Life Sciences (Narilis), Namur University, Namur, Belgium.
[3]Health Unit, Flemish Institute for Technological Research (VITO), Mol, Belgium.
[4]Department of Internal Medicine, Maastricht University Medical Centre (MUMC+), Maastricht, The Netherlands.
[5]Department of Public Health & Primary Care, Occupational and Environmental Medicine, Leuven University, Leuven, Belgium.

*Correspondence:

Prof. Dr. T.S. Nawrot, Centre for Environmental Sciences, Hasselt University, Agoralaan Building D, 3590 Diepenbeek, Belgium. Telephone: 32-11-268382. Fax: 32-11-268299. E-mail: tim.nawrot@uhasselt.be

Table S1. Percentile tables for macrovascular characteristics for children aged 4-6 years.

Macrovascular characteristics	P5	P10	P25	P50	P75	P90	P95
**Age, y**	4.1	4.2	4.3	4.5	4.8	5.1	5.4
**Height, cm**	100.2	102.0	104.4	107.5	111.0	114.3	116.0
**Weight, kg**	15.1	15.9	17.0	18.4	20.3	22.1	23.6
**BMI, kg/m^2**	14.0	14.4	15.1	16.0	16.9	17.8	18.7
**Systolic blood pressure, mmHg**	83.6	88.4	93.4	97.3	102.3	108.6	112.6
**Diastolic blood pressure, mmHg**	43.9	46.0	50.3	53.7	58.3	63.6	67.2
**Heart rate, beats per minute**	74.6	77.8	83.7	89.7	96.7	102.3	107.0
**Carotid intima-media thickness, µm**	382.9	401.0	433.9	486.2	525.8	587.9	604.1
**Pulse wave velocity, m/s**	2.7	2.9	3.2	3.6	4.1	4.7	5.2
**Distensibility coefficient, 10^{-23} /kPa**	23.4	37.0	55.9	71.5	92.0	115.1	137.9
**Young's elastic modulus, kPa**	73.1	90.1	111.2	150.0	186.7	259.6	327.8
**Carotid distension, µm**	460.7	545.1	661.2	778.3	880.2	966.7	1027.8
**Stiffness index β**	2.1	2.6	3.2	3.9	4.8	6.1	7.8
**Compliance coefficient, (mm^2/kPa)**	0.7	1.0	1.3	1.6	1.9	2.3	2.6

Table S2. Percentile tables for macrovascular characteristics for boys and girls.

Macrovascular characteristics	Boys							Girls						
	P5	P10	P25	P50	P75	P90	P95	P5	P10	P25	P50	P75	P90	P95
**Age, y**	4.1	4.2	4.3	4.5	4.8	5.1	5.4	4.1	4.2	4.3	4.5	4.7	5.0	5.2
**Height, cm**	101.5	102.5	104.4	107.5	111.0	114.2	115.0	100.0	101.5	104.4	107.6	110.5	114.9	116.5
**Weight, kg**	15.2	16.0	17.2	18.4	20.3	21.8	23.6	14.8	15.6	17.0	18.4	20.4	22.2	23.3
**BMI, kg/m²**	14.0	14.4	15.2	16.0	16.9	17.6	18.3	14.0	14.4	15.0	16.0	17.0	18.1	18.7
**Systolic blood pressure, mmHg**	83.4	88.1	93.3	97.3	102.6	107.9	113.4	84.0	90.0	93.9	97.3	101.8	109.0	112.0
**Diastolic blood pressure, mmHg**	43.0	45.1	49.1	53.0	57.6	63.3	68.9	44.3	47.0	51.0	54.7	60.1	64.3	66.3
**Heart rate, beats per minute**	72.4	77.7	84.7	89.3	96.3	100.6	107.0	76.3	78.0	82.9	90.0	96.8	102.3	106.3
**Carotid intima-media thickness, µm**	383.6	404.5	441.0	486.8	541.5	590.4	604.6	378.6	396.8	432.6	484.0	521.6	582.5	604.3
**Pulse wave velocity, m/s**	2.6	2.8	3.1	3.6	4.1	4.8	5.5	2.8	3.0	3.2	3.6	4.1	4.6	5.0
**Distensibility coefficient, 10^{-23}/kPa**	33.0	41.1	54.1	72.8	95.9	125.1	152.9	29.0	43.0	60.5	72.1	90.4	106.1	128.3
**Young's elastic modulus, kPa**	64.0	79.8	108.0	152.4	196.0	266.2	348.0	79.5	92.7	112.3	149.0	183.4	237.1	309.8
**Carotid distension, µm**	455.0	533.4	662.5	791.5	889.2	995.9	1055.0	506.2	548.9	645.8	765.3	861.7	913.1	957.5
**Stiffness index β**	1.6	2.5	3.2	4.0	4.8	6.3	8.3	2.4	2.7	3.3	3.9	4.9	5.9	7.3
**Compliance coefficient, (mm^2/kPa)**	0.5	0.9	1.2	1.5	2.0	2.4	2.7	0.8	1.0	1.3	1.6	1.9	2.2	2.5
